# Metabolic Syndrome in People Living with Human Immunodeficiency Virus: An Assessment of the Prevalence and the Agreement between Diagnostic Criteria

**DOI:** 10.1155/2017/1613657

**Published:** 2017-03-14

**Authors:** Kim Anh Nguyen, Nasheeta Peer, Anniza de Villiers, Barbara Mukasa, Tandi E. Matsha, Edward J. Mills, Andre Pascal Kengne

**Affiliations:** ^1^Non-Communicable Diseases Research Unit, South African Medical Research Council, Cape Town 7505, South Africa; ^2^Department of Medicine, University of Cape Town, Cape Town 7935, South Africa; ^3^United Nations Population Fund (UNFPA), Mildmay Uganda, P.O. Box 24985, Lweza, Uganda; ^4^Department of Biomedical Sciences, Faculty of Health and Wellness Science, Cape Peninsula University of Technology, Cape Town 7535, South Africa; ^5^Global Evaluation Science, Vancouver, BC, Canada V6H 3X4

## Abstract

*Objectives.* We determined metabolic syndrome (MetS) prevalence and assessed the agreement between different diagnostic criteria in HIV-infected South Africans. *Method*. A random sample included 748 HIV-infected adult patients (79% women) across 17 HIV healthcare facilities in the Western Cape Province. MetS was defined using the Joint Interim Statement (JIS 2009), International Diabetes Federation (IDF 2005), and Adult Treatment Panel III (ATPIII 2005) criteria. *Results*. Median values were 38 years (age), 5 years (diagnosed HIV duration), and 392 cells/mm^3^ (CD4 count), and 93% of the participants were on antiretroviral therapy (ART). MetS prevalence was 28.2% (95%CI: 25–31.4), 26.5% (23.3–29.6), and 24.1% (21–27.1) by the JIS, IDF, and ATPIII 2005 criteria, respectively. Prevalence was always higher in women than in men (all *p* < 0.001), in participants with longer duration of diagnosed HIV (all *p* ≤ 0.003), and in ART users not receiving 1st-line regimens (all *p* ≤ 0.039). The agreement among the three criteria was very good overall and in most subgroups (all kappa ≥ 0.81). *Conclusions*. The three most popular diagnostic criteria yielded similarly high MetS prevalence in this relatively young population receiving care for HIV infection. Very good levels of agreement between criteria are unaffected by some HIV-specific features highlighting the likely comparable diagnostic utility of those criteria in routine HIV care settings.

## 1. Introduction

The introduction of multiple components antiretroviral therapy (ART) has led to a global decline in AIDS-related mortality among HIV-infected people, with the disease now regarded as a chronic condition. It remains, however, a major contributor to the global burden of disease, with HIV-infected individuals now succumbing to non-AIDS-related comorbidities. These are commonly associated with unhealthy lifestyle behaviours and ageing, similar to the general population, and are a testimony to the success of ART in extending lifespans. Cardiovascular diseases (CVDs) and their cardiometabolic risk factors of hypertension, type 2 diabetes mellitus (hereafter referred to as diabetes), dyslipidaemia, and obesity are rising health priorities in the HIV-infected population [1, 2]. Notably, these cardiometabolic conditions appear to be more prevalent and to occur at younger ages in the HIV-infected compared to noninfected populations [3].

Cardiometabolic diseases are known to frequently cluster with this constellation referred to as the metabolic syndrome (MetS). A diagnosis of MetS is important because it identifies individuals at increased risk for CVDs, diabetes, and all-cause mortality [4]. The pathophysiology of MetS in HIV-infected individuals is thought to be complex with HIV infection per se, prolonged use of ART drugs, and traditional CVD risk factors contributing to the process [5]. Moreover, weight gain may occur following the uptake of ART, with the use of protease inhibitors (PIs) or nucleoside reverse transcriptase inhibitors (NRTIs), in particular, leading to body fat depositions in the abdominal and dorsal regions; this may further contribute to the development of MS among HIV-infected individuals [6].

Considering the possible additional pathways, and thus the subsequent greater risk for the development of MetS in HIV-infected individuals, it is essential to determine the MetS prevalence in this population. According to a recent meta-analysis, the prevalence of MetS in the HIV-infected was 17–31% worldwide, depending on the diagnostic criteria used [7]. Although South Africa has the greatest number of HIV-infected individuals, who account for 17% of the global HIV/AIDS population [8], there is a dearth of literature on the MetS in the local HIV-infected population. Such data is required to guide the appropriate allocation of resources and the development of cost-effective therapeutic strategies and programmes for MetS care in the HIV-infected individuals. Furthermore, it underscores the importance of a holistic approach in the management of HIV-infected individuals. Thus, this study aimed to determine the MetS prevalence in HIV-infected patients receiving care at public healthcare facilities in the Western Cape Province of South Africa using various diagnostic criteria and to assess the agreement between these criteria.

## 2. Research Design and Methods

### 2.1. Study Population and Sampling

A cross-sectional study was conducted between March 2014 and February 2015 among HIV-infected men and women aged 18 years and older, who received care at primary healthcare facilities in the Western Cape. Details of the study method have been described previously [9]. In brief, the participants were sampled from 17 healthcare facilities including 10 facilities in Cape Town and seven in the surrounding rural municipalities using random sampling procedures. Patients who were pregnant or breastfeeding, bedridden, undergoing treatment for cancer, on corticosteroid treatment, unwilling, or unable to give consent were excluded from the study.

The study was approved by the South African Medical Research Council Ethics Committee and conducted in accordance with the principles of the Declaration of Helsinki. Permission to conduct the survey was obtained from the Health Research Office of the Western Cape Department of Health and the selected healthcare facilities.

### 2.2. Data Collection

A team of trained clinicians, nurses, and fieldworkers collected the data using questionnaires, clinical measurements, and biochemical analyses. The data were captured on personal digital assistants (PDAs), using the electronic case report forms with built-in checks for quality control. These were then encrypted at the point of collection and sent via mobile connections to a dedicated server where it was further checked, downloaded, and stored for future use. While the interviews and physical examinations were done on the day of recruitment, blood samples were taken the following day after the participant had fasted overnight.

Sociodemographic data and medical history were obtained using a structured interviewer-administered questionnaire adapted from the World Health Organization's (WHO) STEPwise approach to Surveillance (STEPS) tool. Duration of diagnosed HIV infection, CD4 count, and ART regimens were obtained from participants' records. The 1st-line regimen was a combination of 2 nucleoside reverse transcriptase inhibitors (2NRTIs) and a nonnucleoside reverse transcriptase inhibitor (NNRTI), while the 2nd regimens combined 2 nucleoside reverse transcriptase inhibitors (2NRTIs) and a boosted protease inhibitor (2PIs) [10].

Anthropometric measurements used standardised techniques. Heights and weights were taken with the participants in light clothing and barefooted. Waist circumference (WC) to the nearest centimetre was measured at the level of umbilicus. Blood pressure (BP) was measured on the right arm, using a digital automatic BP monitor (Omron M6 Comfort, Netherlands) after the participant was seated in a resting position for at least five minutes; three measurements were taken three minutes apart whereas the average value of the 2nd and 3rd measurements was used as BP level in the analysis.

Biochemical parameters were analyzed at an ISO 15189 accredited pathology laboratory (PathCare, Reference Laboratory, Cape Town, South Africa). Serum cholesterol and triglycerides were analyzed by enzymatic colorimetric methods; plasma glucose was measured by the hexokinase method; all are implemented using a Beckman Coulter AU 500 spectrophotometer. Insulin concentrations were measured by the chemiluminescence immunoassay method. The homeostatic model assessment of insulin resistance (HOMA-IR) was calculated as the product of insulin (mIU/L) and glucose (mmol/L) by 22.5 [11].

### 2.3. Definitions of MetS

Several international organisations have proposed various definitions for MetS [12]. Those considered in the current study were the European Group for the Study of Insulin Resistance criteria (EGIR 1999) [13], ATPIII 2005 [14], IDF 2005 [15], and JIS 2009 [12], described in Table 1. Both WHO and EGIR definitions have proposed insulin resistance as a prerequisite for diagnosing the MetS. EGIR defines insulin resistance as plasma insulin levels above 75th percentile from nondiabetes populations [13] while WHO recommends the use of hyperinsulinaemic euglycaemic clamp—glucose uptake values below 25th percentile [16]. Because the hyperinsulinaemic euglycaemic clamp and urinary albumin excretion assessments were not undertaken in our study, the WHO criteria were excluded from our analysis [16].

### 2.4. Statistical Analysis

The R statistical software version 3.0.3 (March 6, 2014) was used for statistical analysis. The numerical variables are expressed as means (±SD) or medians (25th–75th percentiles) and categorical variables as counts and percentages. Groups' comparison used the Mann–Whitney *U* test and chi-square test as appropriate. The agreement between the diagnostic criteria for the MetS was assessed with the use of the kappa statistics. Kappa statistics values are interpreted as poor (kappa ≤ 0.2), fair (0.2 < kappa ≤ 0.4), moderate (0.4 < kappa ≤ 0.6), substantial (0.6 < kappa ≤ 0.8), and very good (kappa > 0.8) [17]. In the main analysis, we determined the prevalence of the MetS by the ATPIII 2005, the IDF, and the JIS criteria and assessed the agreement between these three criteria. The criteria were then expanded to include the EGIR criteria in secondary analyses of a subsample of participants with data available on insulin resistance. A *p* value of 0.05 is considered statistically significant.

## 3. Results

### 3.1. Characteristics of the Participants

Of the 831 participants recruited, 748 had complete data on all components of the MetS and are included in the present analyses. As presented in Table 2, 79% (591 participants) of the sample consisted of women. The median age of the participants was 38 years (25th–75th percentiles: 32–44), with men significantly older than women (41 years versus 37 years, *p* < 0.001). The median duration of diagnosed HIV infection was 5 years (25th–75th percentiles: 2–9) with women diagnosed longer than men (5 years versus 4 years, *p* < 0.001). The median CD4 count was 392 cells/mm^3^ (25th–75th percentiles: 240–604) with counts higher in women (410 cells/mm^3^, 253–627) compared to men (272 cells/mm^3^, 193–448), *p* = 0.001. Most participants (93%) were on ART, with the majority being 1st-line ART users (61%) and the distribution of ART regimens differing in men and women (*p* = 0.005). Compared with men, women were more likely to have greater BMI, waist circumference, total cholesterol, low-density lipoprotein cholesterol (LDL-C), high-density lipoprotein cholesterol (HDL-C), and HOMA index (all *p* ≤ 0.012) but had a lower waist-to-hip ratio (WHR), systolic BP, triglycerides (TGs), and fasting plasma glucose (FPG) levels (all *p* ≤ 0.023).

### 3.2. Prevalence of the Metabolic Syndrome by the JIS, IDF, and ATPIII 2005 Criteria

The prevalence of MetS (95%CI) by the JIS, IDF, and ATPIII 2005 criteria was 28.2% (25–31.4), 26.5% (23.3–29.6), and 24.1% (21–27.1), respectively (Table 3). The MetS prevalence was significantly higher in women compared to men across the three criteria (all *p* < 0.001): JIS (31.3% versus 16.6%), IDF (30.5% versus 11.5%), and ATPIII 2005 (26.9% versus 13.4%).

The prevalence of MetS by the HIV-related characteristics is also presented in Table 3. MetS prevalence was higher in participants with ≥5 years' compared with <5 years' duration of diagnosed HIV infection (all *p* ≤ 0.003): JIS (32.6% versus 22.5%), IDF (30.5% versus 21%), and ATPIII 2005 (28.8% versus 17.9%). With regard to ART use, the prevalence of MetS in the 46 participants who were not on ART was 34.8% (21–48.6) by the JIS and 32.6% (19.1–46.2) by the IDF and ATPIII 2005 criteria. This was not significantly different compared to those on ART (all *p* ≥ 0.175). Across the three criteria by ART regimens, participants on 1st-line ART regimens had lower MetS prevalence compared with 2nd line or other regimens, (all *p* ≤ 0.039). There was a trend towards higher MetS prevalence with CD4 count ≥ 392 cells/mm^3^ compared with <392 cells/mm^3^. However, these were not significantly different by any of the three criteria (all *p* ≥ 0.06): JIS (32.6% versus 25.3%), IDF (32.1% versus 24.7%), and ATPIII 2005 (29.4% versus 21.0%).

### 3.3. Concordance between Different MetS Criteria

The concordance between the JIS, IDF, and ATPIII 2005 MetS criteria overall and by subgroups is shown in Table 4. The overall agreement was very good with a kappa of 0.96 (95%CI: 0.93–0.98) between the JIS and IDF criteria, 0.89 (95%CI: 0.86–0.93) between the JIS and ATPIII 2005 criteria, and 0.84 (95%CI: 0.80–0.89) between the IDF and ATPIII 2005 criteria. Similarly, high levels of agreement were found in women, by subgroups of diagnosed HIV infection duration, CD4 count, and ART regimens. The level of concordance in men was very good between the JIS and ATPIII 2005 criteria [kappa 0.88 (0.77–0.98)] but lower between the JIS and IDF criteria [kappa = 0.79 (0.65–0.93)] and the IDF and ATPIII 2005 criteria [0.62 (0.43–0.81)].

### 3.4. Secondary Analyses in Participants with Insulin Resistance Data

In the subgroup of participants with data available on insulin resistance (*N* = 711), the prevalence of MetS and the agreement between the JIS, IDF, and ATPIII 2005 criteria, overall and within subgroups, were mostly similar to those observed in the main analysis (see Table S1 and Table S2 in Supplementary Material available online at https://doi.org/10.1155/2017/1613657). In this subsample, the prevalence of MetS according to the EGIR criteria was as follows: overall: 12.4%; men versus women: 6.7% versus 13.9%, *p* = 0.018; shorter versus longer duration of diagnosed HIV infection: 9.5% versus 15.2%, *p* = 0.022; CD4 count < 392 cells/mm^3^ versus ≥392 cells/mm^3^: 7.9% versus 17.2, *p* = 0.007; and ART regimens: 10.1% (1st line), 19.4% (2nd line), and 14.4% (other regimens) (*p* = 0.051) (Table S1). The agreement between EGIR and the other criteria was at most fair: EGIR versus JIS 0.33 (0.25–0.40); EGIR versus IDF 0.32 (0.24–0.40); and EGIR versus ATPIII 2005 0.38 (0.30–0.46) (Table S2).

## 4. Discussion

In the present study among HIV-infected participants who were mostly on ART, we found that (1) the prevalence of MetS was high based on the JIS, IDF, and ATPIII 2005 criteria but much lower by the EGIR criteria; (2) across the MetS definitions, the prevalence appeared to be higher among women, participants with longer duration of diagnosed HIV infection, and ART users not receiving 1st-line regimens but was mostly unaffected by CD4 count levels; and (3) the agreement between the JIS, IDF, and ATPIII 2005 was very good overall and in most subgroups, while the agreement of the three criteria with EGIR was generally fair.

### 4.1. MetS Prevalence by Different Criteria in the HIV-Infected Population

The prevalence of the MetS in this study by the different criteria was in line with the findings of a recent meta-analysis which revealed that nearly one-third of global HIV-infected populations have the MetS [7]. Compared with other criteria, the JIS identified the most participants with MetS, which was not surprising. The higher thresholds for WC in the ATPIII 2005 and the mandatory use of WC in the IDF criteria likely ruled out the participants diagnosed with MetS by the JIS criteria but who did not qualify for central obesity based on the IDF or ATPIII criteria. Similarly, the presence of insulin resistance and the higher thresholds for TG and FPG, as well as lower thresholds for HDL-C in women in the EGIR criteria, would explain the differences in the magnitude of MetS prevalence between EGIR and the three other set of criteria.

### 4.2. Comparison of MetS Prevalence in the HIV-Infected and General Populations

The MetS prevalence in this study was within the range of 17–46% published in the general population internationally [18–20] and locally [21, 22]. Studies on the epidemiology of MetS in South Africa have provided the prevalent rates of 30.7% in urban black residents of Cape Town [22] and 26.5% in black adults in rural KwaZulu-Natal with the JIS criteria [21]. Using the JIS, IDF, and ATPIII 2001 criteria, and in a much older (mean age of 51 years) and highly obese urban colored population in Cape Town, Erasmus and coworkers found MetS prevalence of 62%, 60.6%, and 55.4%, respectively [23].

The higher prevalence of MetS among women compared to men in this study is consistent with the recent meta-analysis of MetS in people with HIV infection [7], and in line with the prevalence reports of MetS in the general population [21–23]. Notably, the similarities in prevalent MetS between young HIV-infected people and a much older general population suggest the likely comparable risk of developing MetS-related conditions such as CVDs and diabetes in people with HIV infection, but at a much younger age.

### 4.3. MetS Prevalence and HIV-Related Characteristics

Higher prevalence of MetS was found among participants with longer duration of diagnosed HIV infection and ART users who were not on 1st-line regimens. These associations may in part be explained by older age and drug-related toxicity secondary to prolonged use of ART [24] or the 2nd-line regimens that contain protease inhibitors (PIs). Results of clinical trials suggest that PI-based regimens accelerate the progression of MetS, likely via chronic inflammation and incomplete restoration of the immune system after commencing ART [25, 26]. The lack of effect of CD4 count or ART use, mainly with 1st-line regimens, on the presence of MetS reported in this study, mirrors the results of a recent meta-analysis [7]. It is of note however that the effect of CD4 count was explored in a relatively small sample, with a possibility of low statistical power.

### 4.4. Agreement between Sets of Criteria

In the present study, the agreement between JIS, IDF, and ATPIII 2005 criteria was generally good, implying that with the exception of some subgroups, these sets of criteria generally classify the same individuals as having MetS, or ruled out the diagnosis in the same people. This is not surprising since all three criteria are based on the same components although not always with the same threshold for waist circumference. Generally, the agreement between EGIR and other three criteria was at most fair. The key feature explaining the low agreement was the use of insulin resistance as a criterion in the EGIR definition.

Only two studies have previously assessed the agreement between MetS criteria in HIV-infected people, and these have provided findings mostly in line with ours. Ayodele and colleagues reported a very good agreement between JIS and IDF criteria (kappa = 0.88) [27], whereas the agreement between IDF and EGIR was fair (kappa = 0.30) in the report by Cubero and coworkers [28].

The degree of agreement between the JIS, IDF, and ATPIII 2005 criteria in this study is consistent with data from studies conducted in general populations in Africa [21, 23, 29, 30] and other parts of the world [31–33] with the agreement being better in women than in men [29, 30, 33]. Furthermore, the agreement in our sample was unaffected by HIV-specific characteristics, supporting the comparable diagnostic performance of the commonest sets of diagnostic criteria for MetS in a broader population of people with HIV infection and across the continuum of HIV care.

### 4.5. Strengths and Limitations

The present study has some limitations. The relatively fewer men and ART-naïve participants may lead to unstable estimates of MetS prevalence in these subgroups. The absence of an HIV-negative subgroup limits the direct comparison of our findings with those in the general population. In addition, missing data on HIV-related variables did not allow us to perform a regression analysis, which limited the identification of HIV-specific associations with MetS in our study.

Considering that most HIV-related studies in Africa are single-clinic-based, a major strength of this study is the inclusion of multiple healthcare facilities. Moreover, the included healthcare facilities, selected using random sampling methods, were based in both urban and rural areas known to influence MetS prevalence. This allows for the generalizability of the results to other South African HIV-infected population. Furthermore, this study has provided the most comprehensive analysis of the agreement between MetS diagnostic criteria in people with HIV infection.

## 5. Conclusions

This study has reported a high prevalence of MetS according to the three recent criteria in young adult South Africans with HIV infection, and the likely influence of some, but not all, HIV-related characteristics on the estimates. The very good agreement between these sets of criteria suggests that their sequential application is unlikely to explain differences in MetS prevalence in HIV-infected people across settings and time periods, nor would result in substantial mismatch of individuals' MetS status if these criteria were applied interchangeably in routine settings for the purpose of risk screening and reduction. However, previous studies have advised against uncritical application to the general population in Africa, of internationally advocated diagnostic thresholds for some common parameters to these criteria such as WC, as this could lead to unacceptable MetS risk stratification. Extending these investigations to people with HIV infection should be part of future efforts to promote MetS screening in HIV-infected population in Africa using any of the set of criteria applied in the current study.

## Supplementary Material

Supplementary Tables: Table S1. Prevalence (95% confidence interval) of the metabolic syndrome across different definitions and their variations in a subsample of participants (N=711); Table S2. Kappa statistics and 95% confidence interval for the concordance between the JIS, IDF, ATPIII 2005 and EGIR metabolic syndrome criteria presented by gender and HIV-related subgroups (N=711).

## Figures and Tables

**Table 1:** Criteria used for the diagnosis of the metabolic syndrome.

Criteria	EGIR (1999) [13]	ATPIII (2005) [14]	IDF (2005) [15]	JIS (2009) [12]
Compulsory	IR, fasting insulin > 75th percentile	None	WC ≥ 94 cm (men) WC ≥ 80 cm (women)	None
Additional	Any 2 other criteria	Any 3 criteria	Any 2 other criteria	Any 3 criteria
Obesity				
Men	WC ≥ 94 cm	WC ≥ 102 cm	—	WC ≥ 94 cm
Women	WC ≥ 80 cm	WC ≥ 88 cm		WC ≥ 80 cm
Triglycerides	>2.0 mmol/L	≥1.7 mmol/L	≥1.7 mmol/L	≥1.7 mmol/L
HDL-C				
Men	<1.0 mmol/L	<1.03 mmol/L	<1.03 mmol/L	<1.03 mmol/L
Women	<1.0 mmol/L	<1.3 mmol/L or on dyslipidaemia treatment	<1.3 mmol/L or on dyslipidaemia treatment	<1.3 mmol/L or on dyslipidaemia treatment
Blood pressure	≥140/90 mmHg	≥130/85 mmHg or on hypertension treatment	≥130/85 mmHg or on hypertension treatment	≥130/85 mmHg or on hypertension treatment
Glucose	≥6.1 mmol/L	≥5.6 mmol/L or on diabetes treatment	≥5.6 mmol/L or on diabetes treatment	≥5.6 mmol/L or on diabetes treatment

ATPIII: Adult Treatment Panel III; EGIR: European Group for the Study of Insulin Resistance; IDF: International Diabetes Federation; JIS: Joint Interim Statement; HDL-C: high-density lipoprotein cholesterol; IR: insulin resistance; WC: waist circumference.

**Table 2:** Characteristics of the HIV-infected participants.

Characteristics	Total	Men	Women	*p* value
Median (25th–75th percentiles)	*N* = 748	*N* = 157	*N* = 591	
Age (years)	38 (32–44)	41 (35–47)	37 (31–43)	<0.001
Anthropometry				
Body mass index (kg/m^2^)	26.3 (22.1–32)	21.4 (19.8–22.4)	28.3 (23.8–28.9)	<0.001
Waist circumference (cm)	88 (78–98)	79 (74–88)	90 (80–101)	<0.001
Waist-to-hip ratio	0.86 (0.8–0.91)	0.87 (0.84–0.93)	0.85 (0.8–0.9)	<0.001

Blood pressure (mmHg)				
Systolic	117 (107–130)	123.5 (114.5–140)	115 (105.8–127)	<0.001
Diastolic	82 (75–91)	83 (76–94)	81.5 (74.8–89.8)	0.129

Lipid variables (mmol/L)				
Total cholesterol	4.3 (3.7–5.1)	4.2 (3.5–3.8)	4.4 (3.8–5.1)	0.009
LDL-C	2.5 (2.0–3.1)	2.3 (1.7–3.0)	2.5 (2.0–3.1)	0.012
HDL-C	1.3 (1–1.5)	1.2 (1.0–1.5)	1.29 (1.08–1.52)	0.010
Triglycerides	1 (0.7–1.3)	1.12 (0.75–1.27)	0.97 (0.74–1.28)	0.023
Fasting glucose (mmol/L)	5 (4.6–5.4)	5.1 (4.8–5.5)	4.9 (4.6–5.4)	0.010
HOMA-IR	1.36 (0.84–2.24)	0.94 (0.53–1.64)	1.49 (0.93–2.37)	<0.001

HIV-related factors				
HIV duration (years)	5 (2–9)	4 (2–7)	5 (2.5–9)	<0.001
CD4 count (cells/mm^3^)	392 (240–604)	272 (193–448)	410 (253–627)	0.001

Number (%)	*N* = 699	*N* = 149	*N* = 550	
Antiretroviral treatment				0.005
None	46 (6.6)	7 (4.7)	39 (7.1)	
1st line	426 (60.9)	78 (52.3)	348 (63.3)	
2nd line	79 (11.3)	17 (11.4)	62 (11.3)	
Others	148 (21.2)	47 (31.5)	101 (18.3)	

LDL-C: low-density lipoprotein cholesterol; HDL-C: high-density lipoprotein cholesterol; HIV: human immunodeficiency virus; HOMA index: homeostatic model assessment of insulin resistance.

**Table 3:** Prevalence (95% confidence interval) of the metabolic syndrome by the JIS, IDF, and ATPIII 2005 criteria presented by gender and HIV-related subgroups.

Subgroups	JIS	*p* value	IDF	*p* value	ATPIII 2005	*p* value
Gender (*n* = 748)						
Overall	28.2 (25.0–31.4)	<0.001	26.5 (23.3–29.6)	<0.001	24.1 (21.0–27.1)	<0.001
Men	16.6 (10.8–22.4)		11.5 (6.5–16.5)		13.4 (8.1–18.7)	
Women	31.3 (27.6–35.0)		30.5 (26.8–34.2)		26.9 (23.3–30.5)	

HIV duration (*n* = 740)						
Overall	27.8 (24.6–31.1)	0.002	26.1 (22.9–29.2)	0.003	23.7 (20.6–26.7)	0.001
HIV duration < 5 yrs	22.5 (18.1–26.9)		21.0 (16.8–25.3)		17.9 (13.8–21.9)	
HIV duration ≥ 5 yrs	32.6 (27.9–37.2)		30.5 (26.0–35.1)		28.8 (24.3–33.2)	

CD4 count (*n* = 373)						
Overall	29 (24.4–33.6)	0.118	28.4 (23.8–33)	0.115	25.2 (20.8–29.6)	0.060
CD4 count < 392	25.3 (19.0–31.5)		24.7 (18.5–30.9)		21.0 (15.1–26.8)	
CD4 count ≥ 392	32.6 (25.9–39.3)		32.1 (25.4–38.8)		29.4 (22.9–35.9)	

ART use (*n* = 699)						
Overall	28.0 (24.7–31.4)	0.292	26.2 (22.9–29.4)	0.305	24.3 (21.1–27.5)	0.175
No ART	34.8 (21.0–48.6)		32.6 (19.1–46.2)		32.6 (19.1–46.2)	
On ART	27.6 (24.1–31.0)		25.7 (22.4–29.1)		23.7 (20.5–27.0)	
ART regimens		0.017		0.031		0.039
1st line	23.9 (19.9–28.0)		22.5 (18.6–26.5)		20.7 (16.8–24.5)	
2nd line	32.9 (22.5–43.3)		29.1 (19.1–39.1)		30.4 (20.2–40.5)	
Others	35.1 (27.4–42.8)		33.1 (25.5–40.7)		29.1 (21.7–36.4)	

Data are percentage and 95% confidence intervals; ATPIII: Adult Treatment Panel III; EGIR: European Group for the Study of Insulin Resistance; IDF: International Diabetes Federation; JIS: Joint Interim Statement; HIV: human immunodeficiency virus; ART: antiretroviral therapy. Data were missing for some characteristics. For each grouping variable for which data were missing for some participants, the number of participants with valid data is provided (attached to the name of the variable), as well as the overall prevalence of metabolic syndrome in that subsample.

**Table 4:** Kappa statistics and 95% confidence interval for the concordance between the JIS, IDF, and ATPIII 2005 metabolic syndrome criteria presented by gender and HIV-related subgroups (*N* = 748).

Group and subgroup	Criteria	IDF	JIS
Overall (*n* = 748)	IDF	—	0.96 (0.93–0.98)
	ATPIII 2005	0.84 (0.80–0.89)	0.89 (0.86–0.93)
Men	IDF	—	0.79 (0.65–0.93)
	ATPIII 2005	0.62 (0.43–0.81)	0.88 (0.77–0.98)
Women	IDF	—	0.98 (0.96–1.00)
	ATPIII 2005	0.87 (0.83–0.92)	0.89 (0.85–0.93)
HIV duration—overall (*n* = 740)	IDF	—	0.96 (0.93–0.98)
	ATPIII 2005	0.84 (0.80–0.89)	0.89 (0.85–0.93)
HIV duration < 5 yrs	IDF	—	0.96 (0.92–0.99)
	ATPIII 2005	0.81 (0.73–0.89)	0.86 (0.79–0.92)
HIV duration ≥ 5 yrs	IDF	—	0.95 (0.92–0.99)
	ATPIII 2005	0.86 (0.80–0.92)	0.91 (0.87–0.95)
CD4 count—overall (*n* = 373)	IDF	—	0.99 (0.97–1.00)
	ATPIII 2005	0.89 (0.84–0.94)	0.91 (0.86–0.95)
CD4 count < 392 cells/mm^3^	IDF	—	0.99 (0.96–1.00)
	ATPIII 2005	0.86 (0.78–0.95)	0.88 (0.80–0.96)
CD4 count ≥ 392 cells/mm^3^	IDF	—	0.99 (0.96–1.00)
	ATPIII 2005	0.91 (0.85–0.98)	0.93 (0.87–0.98)
Antiretroviral therapy use—overall (*n* = 699)	IDF	—	0.95 (0.93–0.98)
	ATPIII 2005	0.85 (0.81–0.90)	0.90 (0.87–0.94)
No antiretroviral treatment	IDF	—	0.95 (0.86–1.00)
	ATPIII 2005	0.9 (0.77–1.00)	0.95 (0.86–1.00)
On antiretroviral treatment	IDF	—	0.95 (0.93–0.98)
	ATPIII 2005	0.85 (0.80–0.90)	0.90 (0.86–0.94)
1st-line antiretroviral therapy regimen	IDF	—	0.96 (0.93–0.99)
	ATPIII 2005	0.86 (0.80–0.92)	0.91 (0.86–0.95)
2nd-line antiretroviral therapy regimen	IDF	—	0.91 (0.81–1.00)
	ATPIII 2005	0.85 (0.72–0.98)	0.94 (0.86–1.00)
Other antiretroviral therapy regimens	IDF	—	0.95 (0.90–1.00)
	ATPIII 2005	0.81 (0.71–0.91)	0.86 (0.77–0.95)

ATPIII: Adult Treatment Panel III; IDF: International Diabetes Federation; JIS: Joint Interim Statement; HIV: human immunodeficiency virus. Data were missing for some characteristics. For each grouping variable for which data were missing for some participants, the overall agreement between criteria in that subsample is provided.
