# Supplementary material for: Metabolic Syndrome in People Living with Human Immunodeficiency Virus: An Assessment of the Prevalence and the Agreement between Diagnostic Criteria
Source: Int J Endocrinol. 2017 Mar 14;2017:1613657. doi: 10.1155/2017/1613657 (PMC5368417; doi:10.1155/2017/1613657)
Supplement: Supplementary file 1 — Supplementary Tables: Table S1. Prevalence (95% confidence interval) of the metabolic syndrome across different definitions and their variations in a subsample of participants (N=711); Table S2. Kappa statistics and 95% confidence interval for the concordance between the JIS, IDF, ATPIII 2005 and EGIR metabolic syndrome criteria presented by gender and HIV-related subgroups (N=711). [file 1613657.f1.docx]

**Metabolic syndrome in people living with human immunodeficiency virus: an assessment of the prevalence and the agreement between diagnostic criteria**

**Supplementary Tables**

Table S1. Prevalence (95% confidence interval) of the metabolic syndrome across different definitions and their variations in a subsample of participants (N=711)

Table S2. Kappa statistics and 95% confidence interval for the concordance between the JIS, IDF, ATPIII 2005 and EGIR metabolic syndrome criteria presented by gender and HIV-related subgroups (N=711)

**Table S1. Prevalence (95% confidence interval) of the metabolic syndrome across different definitions and their variations in a subsample of participants (N=711)**

| **Subgroups** | **JIS** | **P-value** | **IDF** | **P-value** | **ATPIII-2005** | **P-value** | **EGIR** | **P-value** |
| --- | --- | --- | --- | --- | --- | --- | --- | --- |
| *Gender (n=711)* |  |  |  |  |  |  |  |  |
| Overall | 28.1 (24.8-31.4) | <0.001 | 26.3 (23.1-29.5) | <0.001 | 23.8 (20.6-26.9) | <0.001 | 12.4 (10-14.8) | 0.018 |
| Men | 16.1 (10.2-22) |  | 10.7 (5.8-15.7) |  | 12.8 (7.4-18.1) |  | 6.7 (2.7-10.7) |  |
| Women | 31.3 (27.5-35.2) |  | 30.4 (26.6-34.2) |  | 26.7 (23-30.4) |  | 13.9 (11-16.7) |  |
| *HIV-duration (n=703)* |  |  |  |  |  |  |  |  |
| Overall | 27.7 (24.4-31.1) | 0.002 | 25.9 (26.7-29.1) | 0.003 | 23.3 (20.2-26.5) | 0.001 | 12.5 (10.1-15) | 0.022 |
| HIV-duration<5yrs | 22.3 (17.8-26.8) |  | 20.7 (16.3-25.1) |  | 17.4 (13.3-21.5) |  | 9.5 (6.3-12.6) |  |
| HIV-duration≥5yrs | 32.5 (27.8-37.3) |  | 30.4 (25.7-35.1) |  | 28.5 (24.0-33.1) |  | 15.2 (11.6-18.8) |  |
| *CD4 count (n=358)* |  |  |  |  |  |  |  |  |
| Overall | 29.6 (24.9-34.3) | 0.187 | 29.1 (24.4-33.8) | 0.184 | 25.7 (21.2-30.2) | 0.103 | 12.6 (9.1-16.0) | 0.007 |
| CD4 count<392cells/mm^3^ | 26.4 (19.9-32.9) |  | 25.8 (19.4-32.3) |  | 21.9 (15.8-28) |  | 7.9 (3.9-11.8) |  |
| CD4 count≥392cells/mm^3^ | 32.8 (25.9-39.6) |  | 32.2 (25.4-39.1) |  | 29.4 (22.8-36.1) |  | 17.2 (11.7-22.7) |  |
| *Antiretroviral therapy use (n=663)* |  |  |  |  |  |  |  |  |
| Overall | 28.1 (24.6-31.5) | 0.565 | 26.1 (22.8-29.4) | 0.589 | 24.1 (20.9-27.4) | 0.385 | 12.7 (10.1-15.2) | 0.108 |
| No antiretroviral therapy | 31.8 (18.1-45.6) |  | 29.6 (16.1-43.0) |  | 29.6 (16.1-43.0) |  | 20.5 (9.1-31.8) |  |
| On antiretroviral therapy | 27.8 (24.3-31.3) |  | 25.9 (22.4-29.3) |  | 23.8 (20.4-27.1) |  | 12.1 (9.6-14.7) |  |
| Antiretroviral therapy regimens |  | 0.015 |  | 0.031 |  | 0.032 |  | 0.051 |
| 1^st^ line | 24 (19.9-28.2) |  | 22.6 (18.5-26.6) |  | 20.6 (16.7-24.5) |  | 10.1 (7.1-13) |  |
| 2^nd^ line | 34.7 (23.7-45.7) |  | 30.6 (19.9-41.2) |  | 31.9 (21.2-42.7) |  | 19.4 (10.3-28.6) |  |
| Others | 35.3 (27.3-43.2) |  | 33.1 (25.3-40.9) |  | 28.8 (21.3-36.3) |  | 14.4 (8.6-20.2) |  |

ATPIII, Adult Treatment Panel III; EGIR, European Group for the Study of Insulin Resistance; IDF, International Diabetes Federation; JIS, Joint Interim Statement; HIV, human immunodeficiency virus. Data were missing for some characteristics. For each grouping variable for which data were missing for some participants, the number of participants with valid data is provided (attached to the name of the variable), as well as the overall prevalence of metabolic syndrome in that subsample.

**Table S2. Kappa statistics and 95% confidence interval for the concordance between the JIS, IDF, ATPIII 2005 and EGIR metabolic syndrome criteria presented by gender and HIV-related subgroups (N=711)**

| **Group & Subgroup** | **Criteria** | **ATPIII-2005** | **IDF** | **JIS** |
| --- | --- | --- | --- | --- |
| *Overall (n=711)* |  |  |  |  |
|  | IDF |  |  | 0.95 (0.93-0.98) |
|  | ATPIII 2005 |  | 0.84 (0.79-0.88) | 0.89 (0.85-0.93) |
|  | EGIR | 0.38 (0.30-0.46) | 0.32 (0.24-0.40) | 0.33 (0.25-0.40) |
| *Men* |  |  |  |  |
|  | IDF |  |  | 0.77 (0.62-0.92) |
|  | ATPIII 2005 |  | 0.58 (0.37-0.78) | 0.86 (0.75-0.98) |
|  | EGIR | 0.51 (0.28-0.74) | 0.25 (0.01-0.49) | 0.42 (0.20-0.63) |
| *Women* |  |  |  |  |
|  | IDF |  |  | 0.98 (0.96-1.00) |
|  | ATPIII 2005 |  | 0.87 (0.82-0.91) | 0.89 (0.85-0.93) |
|  | EGIR | 0.36 (0.27-0.44) | 0.32 (0.24-0.40) | 0.31 (0.23-0.39) |
| *HIV duration-overall (n=703)* |  |  |  |  |
|  | IDF |  |  | 0.95 (0.93-0.98) |
|  | ATPIII 2005 |  | 0.83 (0.78-0.88) | 0.88 (0.84-0.9) |
|  | EGIR | 0.39 (0.31-0.48) | 0.33 (0.25-0.41) | 0.34 (0.26-0.41) |
| *HIV-duration<5yrs* |  |  |  |  |
|  | IDF |  |  | 0.95 (0.92-0.99) |
|  | ATPIII 2005 |  | 0.79 (0.71-0.88) | 0.85 (0.77-0.92) |
|  | EGIR | 0.43 (0.29-0.57) | 0.36 (0.23-0.49) | 0.36 (0.23-0.48) |
| *HIV-duration≥5yrs* |  |  |  |  |
|  | IDF |  |  | 0.95 (0.92-0.98) |
|  | ATPIII 2005 |  | 0.85 (0.79-0.91) | 0.91 (0.86-0.95) |
|  | EGIR | 0.36 (0.26-0.47) | 0.30 (0.2-0.41) | 0.32 (0.22-0.41) |
| *CD4 count-overall (n=358)* |  |  |  |  |
|  | IDF |  |  | 0.99 (0.97-1.00) |
|  | ATPIII 2005 |  | 0.89 (0.83-0.94) | 0.90 (0.85-0.95) |
|  | EGIR | 0.32 (0.21-0.44) | 0.28 (0.17-0.38) | 0.28 (0.18-0.39) |
| *CD4 count<392* |  |  |  |  |
|  | IDF |  |  | 0.99 (0.96-1.00) |
|  | ATPIII 2005 |  | 0.86 (0.77-0.95) | 0.88 (0.80-0.96) |
|  | EGIR | 0.30 (0.13-0.46) | 0.24 (0.09-0.39) | 0.24 (0.09-0.38) |
| *CD4 count≥392* |  |  |  |  |
|  | IDF |  |  | 0.99 (0.96-1.00) |
|  | ATPIII 2005 |  | 0.91 (0.84-0.97) | 0.92 (0.86-0.98) |
|  | EGIR | 0.33 (0.18-0.48) | 0.29 (0.14-0.44) | 0.31 (0.17-0.45) |
| *Antiretroviral therapy use-overall (n=663)* |  |  |  |  |
|  | IDF |  |  | 0.95 (0.92-0.98) |
|  | ATPIII 2005 |  | 0.84 (0.80-0.89) | 0.90 (0.86-0.94) |
|  | EGIR | 0.40 (0.32-0.48) | 0.35 (0.27-0.43) | 0.35 (0.28-0.43) |
| *No antiretroviral therapy* |  |  |  |  |
|  | IDF |  |  | 0.95 (0.84-1.00) |
|  | ATPIII 2005 |  | 0.89 (0.74-1.00) | 0.95 (0.84-1.00) |
|  | EGIR | 0.52 (0.24-0.80) | 0.40 (0.10-0.70) | 0.48 (0.20-0.76) |
| *1^st^ line antiretroviral therapy regimen* |  |  |  |  |
|  | IDF |  |  | 0.96 (0.93-0.99) |
|  | ATPIII 2005 |  | 0.86 (0.79-0.92) | 0.90 (0.85-0.95) |
|  | EGIR | 0.40 (0.28-0.51) | 0.36 (0.25-0.47) | 0.37 (0.26-0.48) |
| *2^nd^ line antiretroviral therapy regimen* |  |  |  |  |
|  | IDF |  |  | 0.91 (0.80-1.00) |
|  | ATPIII 2005 |  | 0.84 (0.70-0.97) | 0.94 (0.85-1.00) |
|  | EGIR | 0.32 (0.09-0.56) | 0.34 (0.11-0.58) | 0.28 (0.06-0.51) |
| *Other antiretroviral therapy regimens* |  |  |  |  |
|  | IDF |  |  | 0.95 (0.90-1.00) |
|  | ATPIII 2005 |  | 0.80 (0.69-0.91) | 0.85 (0.76-0.94) |
|  | EGIR | 0.38 (0.21-0.55) | 0.28 (0.12-0.44) | 0.29 (0.14-0.44) |

ATPIII, Adult Treatment Panel III; EGIR, European Group for the Study of Insulin Resistance; IDF, International Diabetes Federation; JIS, Joint Interim Statement; HIV, human immunodeficiency virus. Data were missing data for some characteristics. For each grouping variable for which data were missing for some participants, the overall agreement between criteria in that subsample is provided.
